# Plastome Characterization and Phylogenomics of East Asian Beeches with a Special Emphasis on *Fagus multinervis* on Ulleung Island, Korea

**DOI:** 10.3390/genes11111338

**Published:** 2020-11-12

**Authors:** JiYoung Yang, Koji Takayama, Jin-Suk Youn, Jae-Hong Pak, Seung-Chul Kim

**Affiliations:** 1 Research Institute for Dok-do and Ulleung-do Island, Department of Biology, School of Life Sciences, Kyungpook National University, 80 Daehak-ro, Buk-gu, Gyeongsangbuk-do, Daegu 41566, Korea; whity@daum.net (J.Y.); okita0522@naver.com (J.-S.Y.); 2 Department of Botany, Graduate School of Science, Kyoto University, Oiwake-cho, Kitashirakawa, Sakyo-ku, Kyoto 606-8502, Japan; takayama.koji.3x@kyoto-u.ac.jp; 3 Department of Biological Sciences, Sungkyunkwan University, 2066 Seobu-ro, Gyeonggi-do, Suwon 16419, Korea

**Keywords:** plastome, eastern asia beeches, *Fagus multinervis*, Ulleung Island, Fagaceae

## Abstract

Beech trees of the genus *Fagus* (Fagaceae) are monoecious and distributed in the Northern Hemisphere. They represent an important component of mixed broad-leaved evergreen–deciduous forests and are an economically important source of timber. Despite their ecological and economical importance, however, little is known regarding the overall plastome evolution among *Fagus* species in East Asia. In particular, the taxonomic position and status of *F*. *multinervis*, a beech species endemic to Ulleung Island of Korea, remains unclear even today. Therefore, in this study, we characterized four newly completed plastomes of East Asian *Fagus* species (one accession each of *F*. *crenata* and *F*. *multinervis* and two accessions of *F*. *japonica*). Moreover, we performed phylogenomic analyses comparing these four plastomes with *F. sylvatica* (European beech) plastome. The four plastomes were highly conserved, and their size ranged from 158,163 to 158,348 base pair (bp). The overall GC content was 37.1%, and the sequence similarity ranged from 99.8% to 99.99%. Codon usage patterns were similar among species, and 7 of 77 common protein-coding genes were under positive selection. Furthermore, we identified five highly variable hotspot regions of the *Fagus* plastomes (*ccsA*/*ndhD*, *ndhD/psaC*, *ndhF*/*rpl32*, *trnS*-GCU/*trnG*-UCC, and *ycf1*). Phylogenetic analysis revealed the monophyly of *Fagus* as well as early divergence of the subgenus *Fagus* and monophyletic *Engleriana.* Finally, phylogenetic results supported the taxonomic distinction of *F. multinervis* from its close relatives *F. engleriana* and *F. japonica*. However, the sister species and geographic origin of *F. multinervis* on Ulleung Island could not be determined.

## 1. Introduction

Genus *Fagus* L. (Fagaceae), commonly distributed in the Northern Hemisphere, is an ecologically and economically important tree lineage of broad-leaved deciduous-evergreen forests of North America and East Asia as well as the most abundant broad-leaved tree genus in Europe and West Asia [1,2,3,4]. With the center of diversity in East Asia, *Fagus* comprises 10 monoecious broad-leaved deciduous tree species within two subgenera, namely *Engleriana* (three species) and *Fagus* (seven species) [1,5]. While subgenus *Engleriana* is restricted to East Asia (China, Korea, and Japan), *Fagus* is distributed almost throughout the Northern Hemisphere. *Fagus japonica* Maxim. and *F. okamotoi* Shen [5] of subgenus *Engleriana* are endemic to Japan, while *F. engleriana* Seemen is distributed in China; the taxonomic entity of *F. okamotoi* is uncertain in Japan and it is not currently recognized as a distinct species. Of the seven species of subgenus *Fagus*, *F. sylvatica* L. is widely distributed in Europe and southwestern Asia and *F. grandifolia* Ehrh. in eastern North America and Mexico [2]. Meanwhile, some species are geographically restricted to specific regions, such as *F. crenata* Blume to Japan, *F. longipetiolata* Seemen and *F. lucida* Rehder and Wilson to China, and *F. chienii* Cheng to western China. *F. hayatae* Palibin is distributed in a disjunct manner in Mainland China and Taiwan. Moreover, *F. okamotoi* has been recorded from few localities in Japan, while *F. chienii* is recorded from a single locality in western China [2]. In addition, former distinct species but current synonyms of *F. longipetiolata* show restricted geographical distribution; for instance, *F. brevipetiolata* Hu is recorded from few localities in China and *F. bijiensis* C.F. Wei & Y.Y. Chang and *F. tientaiensis* T.N. Liou are recorded from a single locality in western China [1,2,5].

Based on numerous fossil records (see references in Denk [1] and Renner et al. [6]), *Fagus* has been speculated to have already existed in the early Cenozoic in the northern Pacific Basin, extending to the Axel Heiberg Island and western Greenland and further spreading westward to Central Asia and Europe during the Oligocene. Based on 53 fossil records and nuclear sequences of nine *Fagus* species, Renner et al. [6] constructed a fossilized birth-death model to estimate the divergence time of major lineages within this genus. The model revealed that the crown group originated in the early Eocene, nearly 53 million years ago (Ma); *F. grandifolia* (America) diverged 44 Ma; *F. sylvatica* (western Eurasia) diverged from *F. crenata* (Japan) nearly 23 Ma; and *F. sylvatica* (Central Europe) diverged from *F. orientalis* (eastern Mediterranean; now treated as a conspecific of *F. sylvatica*) nearly 9 Ma. In addition to molecular dating, several phylogenetic analyses have been performed to infer species relationships within the genus. For instance, the non-monophyly of subgenus *Engleriana* and overall diverse species relationships within the genus have been demonstrated [7,8]. Meanwhile, a morphological study has revealed the monophyly of subgenus *Engleriana*, which is either deeply nested within or reciprocally monophyletic with subgenus *Fagus* [1]. Another study based on nuclear internal transcribed spacer (ITS) region sequences and morphological data [2] showed the early divergence of Eurasian species and paraphyly of subgenus *Fagus*. Given this tree topology, subgenus *Engleriana* has been inferred to be nested within subgenus *Fagus*. Morphological data have also suggested the early divergence of *F. hayatae* and *F. longipeiolata* from Taiwan and China and identified two intercontinental disjunct distributed taxa, namely *F. crenata* (Japan) and *F. sylvatica* (Europe) as well as *F. grandifolia* (eastern North America) and *F. engleriana*/*F. japonica*/*F. okamotoi* (East Asia). Based on nuclear ITS region and *LEAFY* sequence data, Renner et al. [6] demonstrated the early divergence of monophyletic subgenus *Engleriana* (*F. engleriana* and *F. japonica*) and its sister relationship to monophyletic subgenus *Fagus*. Furthermore, Oh et al. [9] reconstructed the phylogeny of *Fagus* based on both nuclear (*LEAFY*) and chloroplast (cpDNA) sequence data but yielded largely unsupported and conflicting topologies. Specifically, the tree based on cpDNA sequences suggested unresolved relationships among the lineages of *F. grandifolia*, *F. sylvatica*, *F. crenata*/*F. japonica*, and *F. engleriana* (including *F. multinervis*)/*F. longipetiolata*/*F. japonica*. Meanwhile, the tree based on *LEAFY* sequences identified clades containing *F. engleriana* (including *F. multinervis*) and *F. japonica* as well as *F. lucida*/*F. longipetiolata*. Therefore, a large-scale phylogenomic study is warranted to resolve phylogenetic relationships of species within the genus as well as to infer hybridization and introgression.

Similar to several other taxonomically controversial taxa (e.g., *F. orientalis*, *F. brevipetiolata*, *F. bijiensis*, and *F. tientaiensis*, among others), the phylogenetic position and species delimitation of *F. multinervis* have remained unclear for several decades. In the Korean Peninsula, a single *Fagus* species, *F. multinervis*, occurs on Ulleung Island but its taxonomic status and relationship to other *Fagus* species remains controversial. Ulleung Island (with the estimated age of ca. 1.8 Ma) is an oceanic and a volcanic island located at about 130 km from the eastern coast of the Korean Peninsula. As a dominant tree in mixed evergreen-deciduous forests at elevations ranging from 400 to 940 m, *F. multinervis* represents an ecologically, economically and culturally important component of the island [9]. Considering leaf, anther, and pollen characteristics, *F. multinervis* was considered an ecological variant of *F. japonica*, which is endemic to central and southern Japan [10]. Meanwhile, based on cupule characteristics, *F. multinervis* has been more frequently and recently treated as a synonym of *F. engleriana*, implying disjunct distribution in western and eastern China as well as Ulleung Island in Korea [1,2,5,11]. However, *F. multinervis* has also been proposed as a taxonomically distinct species endemic to Ulleung Island [12,13,14,15]. Specifically, Oh et al. [9] demonstrated the monophyly of *F. multinervis* and its unresolved relationships with *F. engleriana* and *F. japonica*. They supported the recognition of *F. multinervis* as a distinct species endemic to Ulleung Island and its potential hybrid origin based on the topological incongruence. Nonetheless, despite several aforementioned previous reports, the sister relationships and geographical origin of *F. multinervis* require additional evidence.

Recently, whole plastome phylogenomic analyses have gained popularity due to unique characteristics of plastomes and technical advantages [16]. Owing to high structural conservation and slow evolutionary rate, plastid phylogenomics has significantly advanced our understanding of unresolved high- or deep-level relationships among angiosperms [17,18,19,20,21]. In addition, plastome sequencing has revealed considerable variation within and between plant species [16]. Therefore, such information is particularly fundamental to provide a greater resolution and stronger support at lower taxonomic levels [22,23,24,25,26,27,28,29]. Recently, several studies have reported the whole plastome sequences of *Fagus* and related genera, such as *Quercus* and *Castanea*, in Fagaceae [30,31,32,33,34]. Previous phylogenetic studies of *Fagus* were based primarily on nuclear ITS region and *LEAFY* intron and rarely on plastid coding or noncoding regions, which are either highly variable or highly conserved, providing unsupported congeneric relationships [2,9]. Therefore, recent characterization studies have been particularly helpful to understand plastome organization and structure in congeners of *Fagus* as well as to determine their phylogenetic relationships. To clarify the phylogenetic position and taxonomic status of *F. multinervis* endemic to Ulleung Island, two accessions of this species were sequenced, which revealed intraspecific variation [31,33]. However, these studies, which did not include *F. japonica* endemic to Japan, suggested that *F. engleriana* is sister to two accessions of *F. multinervis* and that *F. crenata*, another species endemic to Japan, is sister to the clade containing *F. sylvatica*/*F. engleriana*/*F. multinervis*. To determine the precise phylogenetic position of *F. multinervis* based on plastome data, *F. japonica* must be included in the analysis. Furthermore, structural variation and molecular evolution of plastomes within *Fagus* have been addressed only to a limited extent based on two species (*F. crenata* and *F. engleriana*) [32,34], which cannot clarify the whole scenario of plastome evolution within this genus.

To this end, in the present study, we characterized the first two plastome sequences of *F. japonica* and the second accession of *F. crenata*, both of which are endemic to Japan. We also sequenced an additional accession of *F. multinervis* from the northern part of Ulleung Island and compared it with two previously reported accessions from the eastern and southeastern parts of the island. Furthermore, we performed comparative analyses across nine plastomes in two subgenera, namely *Fagus* (*F. crenata* and *F. sylvatica*) and *Engleriana* (*F. engleriana*, *F. japonica*, and *F. multinervis*). We aimed to (1) characterize the plastome structure and evolution within *Fagus*, (2) gain insight into the phylogenetic position of *F. multinervis* on Ulleung Island, and (3) identify useful chloroplast markers, including mutation hotspots, to construct strongly supported and highly resolved phylogenetic trees of *Fagus* species. Given the limited samples and geographic coverage used in this study, we cautiously interpret our results for the first two objectives, requiring further evidence from a large-scale plastid phylogenomic study and independent nuclear loci data.

## 2. Materials and Methods

### 2.1. Plant Sampling, DNA Isolation, and Plastome Sequencing and Annotation

We sampled one accession of *F. crenata* and two accessions of *F*. *japonica* from Sasari, Nantan, Kyoto Prefecture, Japan (35°16′39.4″ N 135°43′50.2″ E) and one accession of *F*. *multinervis* from Buk-myeon, Ulleung Island, Korea (37°30′57 N 130°52′10 E). A previously sequenced accession of *F. crenata* (NC041252) was collected from Daisengen Peak on the northern island Hokkaido, Japan (41.616° N, 140.1333° E) [32]. The accession sequenced in this study represents a sample from southern Japan. The *F. multinervis* accession sequenced in this study was collected from the northern part of Ulleung Island (~400 m elevation), while the two previously sequenced accessions [31,33] were collected from the eastern (37°30′ N, 130°54′ E, 216 m elevation) and southeastern (37°29′19.0″ N, 130°53′15.9″ E, 471 m elevation) parts of the island. Voucher specimens are deposited in the Ha Eun Herbarium of Sungkyunkwan University, Korea.

Fresh leaves were collected and dried with silica gel before DNA extraction. Total DNA was extracted using the DNeasy Plant Mini Kit (Qiagen, Carlsbad, CA, USA) and sequenced with Illumina HiSeq 4000 (Illumina, Inc., San Diego, CA, USA), yielding 150 bp paired-end read length, at Macrogen Co. (Seoul, Korea). The resulting paired-end reads were assembled de novo using Velvet v1.2.10 with multiple k-mers [35]. tRNAs were confirmed using tRNAscan-SE [36]. Sequences were annotated using Geneious R10 [37] and deposited in GenBank [*F*. *crenata* (MT762292), *F*. *japonica7-1* (MT762294), *F*. *japonica10-1* (MT762295), and *F*. *multinervis* (MT762296)]. Annotated sequence files in the GenBank format were used to draw a circular map with OGDRAW v1.2 [38].

### 2.2. Comparative Plastome Analysis

Using the Shuffle-LAGAN mode [39] of mVISTA [40], six complete plastomes of *Fagus* species were compared: one plastome each of *F*. *crenata*, *F*. *engleriana*, *F*. *multinervis*, and *F*. *sylvatica* and two plastomes of *F*. *japonica*. Sequences of the six *Fagus* plastomes were aligned using the back-translation approach with MAFFT ver.7 [41] and manually edited with Geneious R10 [37]. Using DnaSP 6.10 [42], sliding window analysis with a step size of 200 bp and window length of 800 bp was performed to determine nucleotide diversity (*Pi*) of the plastomes. Codon usage frequency was calculated using MEGA 7 [43] based on the relative synonymous codon usage (RSCU) value [44], which is a simple measure of non-uniform usage of synonymous codons in a coding sequence. The DNA code used by bacteria, archaea, prokaryotic viruses, and chloroplast proteins was used [45]. Protein-coding genes were run using the PREP suite [46] with 35 reference genes and a cut off value of 0.8 to predict possible RNA editing sites in five *Fagus* plastomes that were newly reported in the present study. Analyses based on complete plastomes and concatenated sequences of 77 common protein-coding genes of the studied *Fagus* species were performed using MAFFT ver.7 [41] in Geneious R10 (Kearse et al., 2012). A maximum likelihood (ML) phylogenetic tree was constructed using IQ-TREE 1.4.2 [47]. To evaluate natural selection pressure on the protein-coding genes of the five *Fagus* plastomes, a site-specific model was developed using EasyCodeML [48] with the CODEML algorithm [49]. Seven codon substitution models (M0, M1a, M2A, M3, M7, M8, and M8a) were constructed and compared to detect positively selected sites based on likelihood ratio test (LRT).

### 2.3. Phylogenetic Analysis

For phylogenetic analysis, complete plastome sequences of 17 representative species of Fagaceae were aligned with MAFFT ver.7 [41] in Geneious R10 [37]: two *Castanea* species, including *C*. *henryi* (NC033881) and *C*. *pumila* (KM360048); five *Quercus* species, including *Q*. *aquifolioides* (NC026913), *Q*. *spinosa* (NC026907), *Q*. *rubra* (NC020152), *Q*. *tarokoensis* (NC036370), and *Q*. *variabillis* (NC031356); four *Fagus* species, including *F*. *crenata* (NC041252), *F*. *engleriana* (NC036929), *F*. *multinervis* (MK518070), and *F*. *sylvatica* (NC041437); one *Betula* species, namely *B. nana* (NC033978). ML analysis based on the best-fit model of “K3Pu+F+R3” was conducted with IQ-TREE 1.4.2 [47]. *B. nana* was used as the outgroup, and non-parametric bootstrap analysis was performed with 1000 replicates.

## 3. Results

### 3.1. Genome Size and Characteristics

**Figure 1:**
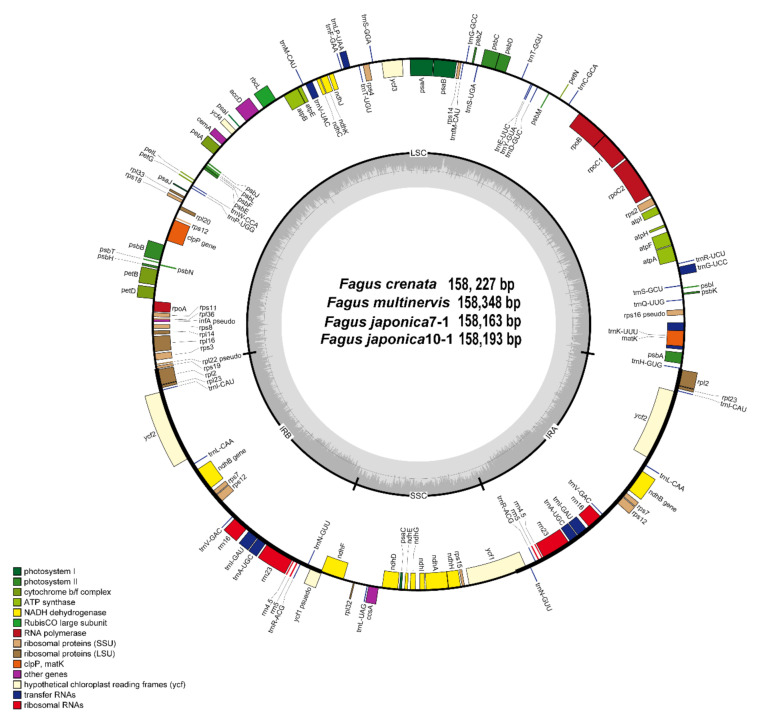
The four *Fagus* plastomes in Fagaceae. The genes located outside of the circle are transcribed clockwise, while those located inside are transcribed counterclockwise. The gray bar area in the inner circle denotes the guanine-cytosine (GC) content of the genome, whereas the lighter gray area indicates the adenosine-thymine (AT) content of the genome. Large single copy, small single copy, and inverted repeat are indicated with LSC, SSC, and IR, respectively.

The plastomes of four accessions of East Asian *Fagus* species were newly characterized, including the plastome of *F*. *japonica* for the first time. The size of complete plastome sequences ranged from 158,348 (*F*. *multinervis*) to 158,163 bp (*F*. *japonica7-1*) (Table 1). The plastomes were highly conserved, with no structural variations or content rearrangements (Figure 1). The four plastomes of East Asian *Fagus* species contained 131 genes, including 82 protein-coding, 8 rRNA, and 37 tRNA genes. Consistent with previous reported values for *Fagus* plastomes (35.5% for *F*. *crenata* [32]; 37.0% for *F*. *engleriana* [34]; and 37.1% for *F*. *multinervis* [31,33]), the overall GC content was 37.1% (Table 1). Moreover, 17 genes were duplicated in the inverted repeat (IR) regions, including 7 tRNA, 4 rRNA, and 6 protein-coding genes. A total of 15 genes (*ndhA*, *ndhB*, *petB*, *petD*, *rpl2*, *rpl16*, *rpoC1*, *rps16*, *rps12*, *trnA*-UGC, *trnG*-UCC, *trnI*-GAU, *trnK*-UUU, *trnL*-UAA, and *trnV*-UAC) contained 1 intron, while 2 genes (*clpP* and *ycf3*) contained 2 introns.

Partial *ycf1* (1113–1131 bp) was located in the IRb/SSC junction region, and complete *ycf1* (5670–5688 bp) was located in the IR region at the SSC/IRa junction. Similar to those of *F*. *engleriana* [34], three protein-coding genes, namely *infA*, *rps16*, and *rpl22*, of the four East Asian *Fagus* plastomes were pseudogenes.

**Figure 2:**
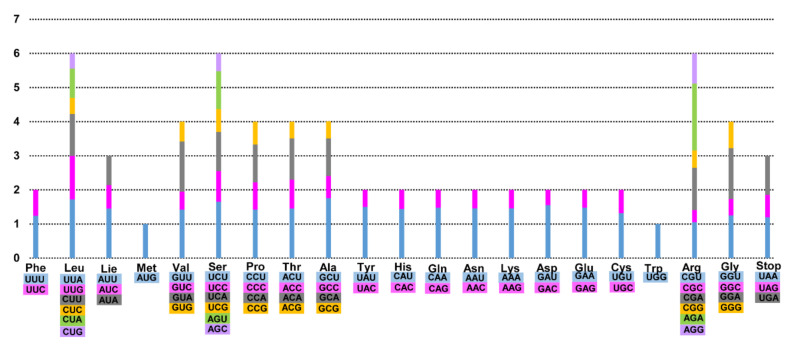
Codon distribution and Relative Synonymous Codon Usage (RSCU) in five *Fagus* plastomes. The RSCU values are represented on the y-axis, while the codon families for each amino acid are denoted on the x-axis.

Codon usage frequency of the five complete East Asia *Fagus* plastomes was calculated using sequences of protein-coding and tRNA genes (Figure 2). Average codon usage frequency ranged from 25,748 (*F*. *crenata*) to 26,053 (*F*. *multinervis*), although the distribution of codon types was consistent. Codon usage in the four East Asian *Fagus* plastomes was biased toward high RSCU values of U and A at the third codon position.

**Figure 3:**
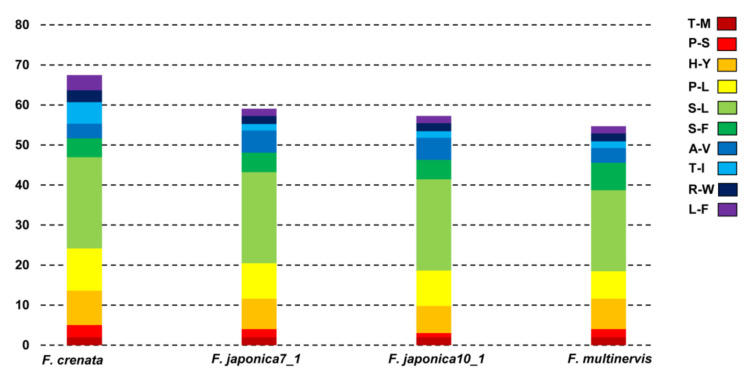
Amino acid changes in potential RNA editing sites of the chloroplast genomes of five *Fagus*. Color bricks indicate RNA editing effect. T-M: Threonine to Methionine, P-S: Proline to Serine, H-Y: Histidine to Tyrosine, P-L: Proline to Leucine, S-L: Serine to Leucine, S-F: Serine to Phenylalanine, A-V: Alanine to Valine, T-I: Threonine to lsoleucine, R-W: Arginine to Tryptophan, L-F: Leucine to Phenylalanine.

Prediction of RNA editing sites in the five East Asian *Fagus* species indicated 75 sites with the same cut-off value in 23 of the 35 protein-coding genes (Table S1). These included photosynthesis-related genes (*atpA*, *atpF*, *atpI*, *ndA*, *ndhB*, *ndhD*, *ndhF*, *ndhG*, *petB*, *psaI*, *psbE*, and *psbF*), self-replication-related genes (*rpoA*, *rpoB*, *rpoC2*, *rps2*, *rps14*, and *rps16*), and others (*accD*, *clpP*, *ccsA*, and *matK*). The *ndhF* in *F*. *multinervis* showed a significantly higher frequency of RNA editing sites (8 sites) than in other species. Moreover, *rpoC2* in *F*. *crenata* showed the highest frequency of RNA editing sites (13 in *F*. *crenata* vs. 3 in others). In *F. japonica* and *F. multinervis*, *ndhB* was predicted to contain the highest number of potential editing sites (10 sites), followed by *ndhD* (seven sites). All editing sites showed base transition from C to T, and the most frequent conversion was serine to leucine (Figure 3). Consequently, amino acids with hydrophobic chains (isoleucine, leucine, methionine, phenylalanine, tryptophan, and valine) were formed in 82.3% of the 23 RNA editing sites.

To assess intraspecific variations in three East Asian *Fagus* species, we compared four newly completed *Fagus* plastomes with previously reported ones (*F*. *crenata*, NC041252; *F*. *multinervis*, MK518070 and MN894556). The plastome of *F. crenata* (158,227 bp) from southern Japan known to have chlorotype “BF” [50] was compared with a previously reported plastome from northern Japan (NC041252, 158,372 bp) known to have chlorotype “A.” The sequences of two intraspecific plastomes shared 99.8% identity, with 59 indels and 45 substitutions. Moreover, 11 protein-coding genes (*atpB*, *atpF*, *matK*, *ndhD*, *petA*, *rpl16*, *rpoB*, *rpoC2*, *rps16*, and *ycf1*) were polymorphic, with 8 indels and 9 substitutions. Of these, *rpoB* and *petA* harbored a single nonsynonymous substitution. In addition, 51 indels and 34 substitutions were detected in intergenic regions in the two *F*. *creanata* plastomes. Specifically, the intergenic region *trnE*-UUU/*trnT*-GGU/*psbD* harbored 33 indels and 26 substitutions.

Two newly sequenced accessions of *F*. *japonica* in the present study shared 99.98% sequence identity, with three indels and one substitution. The total lengths of the two plastomes of *F*. *japonica* 7-1 and *F*. *japonica*10-1 were 158,163 and 158,193 bp, respectively. We also compared three plastomes of *F*. *multinervis* from Ulleung Island, which shared 99.99% sequence identity. The newly assembled plastome of *F*. *multinervis* in this study was identical to the accession MK518070 (158,348 bp) sampled from the eastern part of the island. Compared to the other accession, MN894556, sampled from the southeastern part of the island, a frameshift with one base-pair insertion (T) was detected in *atpB*, resulting in a shorter gene with early termination. In addition, as previously reported, one synonymous substitution in *psbM* and one nonsynonymous substitution in *ccsA* were detected.

### 3.2. Comparative Analysis of Genome Structure

**Figure 4:**
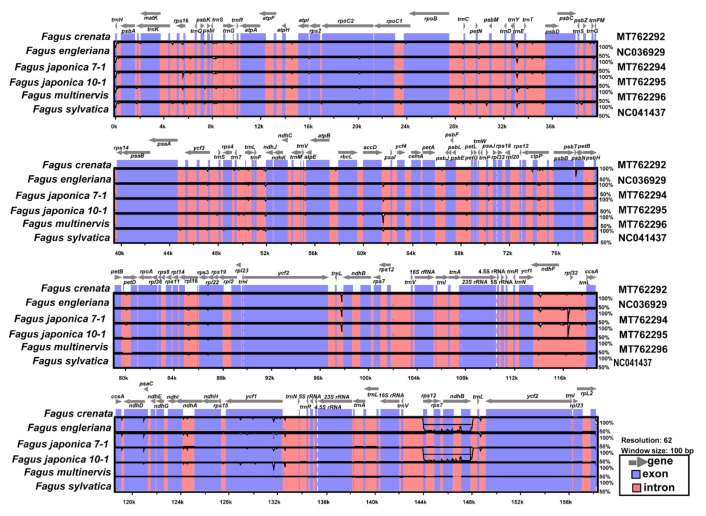
Visualization of alignment of the six *Fagus* plastome sequences of Fagaceae species. Vertical scale indicates the percent identity from 50% to 100%. Coding and non-coding regions are in blue and pink, respectively. Gray arrows above the alignment indicate the position and direction of each gene.

Five complete plastome sequences of East Asian *Fagus* species and one European *Fagus* (*F*. *sylvatica*) were plotted using mVISTA [40], with the annotated *F*. *crenata* plastome as a reference (Figure 4). As expected, the large single-copy (LSC) region was the most divergent, and the two IR regions were highly conserved. Moreover, the non-coding regions were more divergent and variable than the coding regions. Overall, all *Fagus* plastomes showed high sequence similarity (i.e., 99.6% sequence identity; 157,284 bp identical sites) to the *F*. *crenata* plastome.

**Figure 5:**
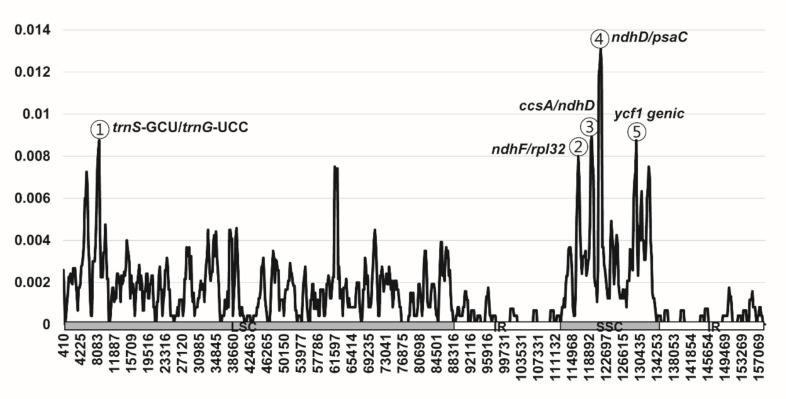
Sliding window analysis of the six whole-chloroplast genomes of *Fagus* species. X-axis: position of the window midpoint, Y-axis: nucleotide diversity within each window.

Sliding window analysis using DnaSP [42] revealed highly variable regions in *Fagus* plastomes (Figure 5). When plastomes of five East Asian and one European *Fagus* accessions were compared, average *Pi* over the whole plastome was 0.0016. The *ndhD/psaC* intergenic region was the most variable region, with a *Pi* value of 0.01308. Moreover, highly variable regions included three other intergenic regions, namely *trnS*-GCU/*trnG*-UCC (*Pi* = 0.00875), *ccsA*/*ndhD* (*Pi* = 0.00892), and *ndhF*/*rpl32* (*Pi* = 0.008), and one *ycf1* genic region (*Pi* = 0.00875). Overall, five highly variable regions with a *Pi* value greater than 0.008 were identified in six *Fagus* plastomes.

The site-specific model developed using EasyCodeML [48] with the CodeML algorithm [49] identified positively selected genes among six *Fagus* plastomes (Table 2). Seven conserved genes across *Fagus* plastomes were predicted to be under positive selection, with significant LRT *p* values. Moreover, in the six comparison groups, 70 of the 77 genes had an average Ka/Ks ratio below 1, suggesting that these genes were subjected to strong purifying selection in the *Fagus* plastomes. The remaining seven genes had an average Ka/Ks ratio greater than 1, suggesting that these genes were positively selected in the six *Fagus* plastomes. These genes include two photosynthesis-related genes encoding NADH-dehydrogenase subunits (*ndhD* and *ndhJ*), two genes encoding DNA-dependent RNA polymerase (*rpoB* and *rpoC2*), one self-replication-related gene encoding the ribosomal small subunit (*rps16*), and two unknown genes (*ycf1* and *ycf2*). Based on the M8 model, *ycf1* had the highest number of positive sites (9 sites), followed by rpoC2 (2 sites), while the other five genes had only number of positive sites (9 sites), followed by *rpoC2* (2 sites), while the other five genes had only one positive site.

### 3.3. Phylogenetic Analysis

**Figure 6:**
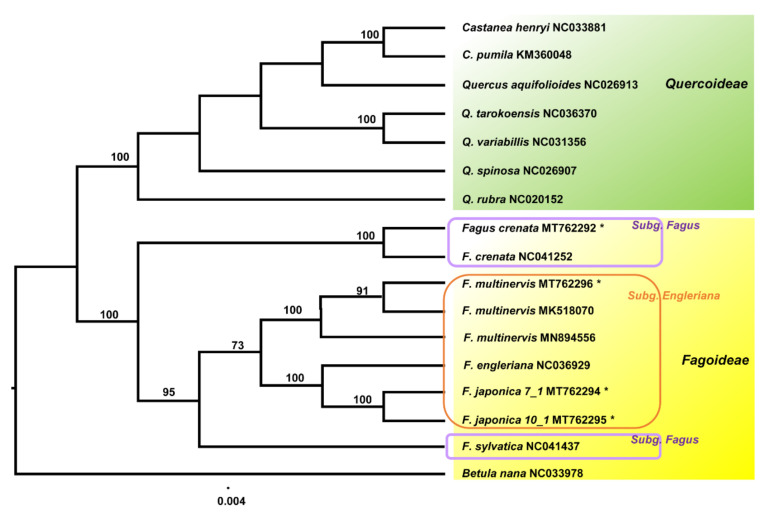
Maximum likelihood tree inferred from 17 representative taxa of Fagaceae. Bootstrap values based on 1000 replicates are shown on each node. Asterisk (*) represents the newly assembled plastomes of *Fagus* (Fagaceae) in this study.

ML of the best-fit model of “K3Pu+F+R3” suggested well-resolved phylogenetic relationships within Fagaceae (Figure 6). Phylogenetic analysis of 16 representative plastomes of Fagaceae supported the monophyly of *Fagus* (100% bootstrap support (BS)) as well as the sister relationship between *Fagus* and the clade containing *Castanea* and *Quercus* (weak support, <60% BS). Within the subfamily Quercoideae, the ML tree suggested that monophyletic *Castanea* is deeply nested within *Quercus*, making it paraphyletic. Within *Fagus*, the plastome phylogenomic tree suggested that Japanese *F. crenata* diverged first, followed by European *F. sylvatica*. Based on three accessions, *F. multinervis* endemic to Ulleung Island is monophyletic (100% BS), whereas *F. engleriana* and the newly sequenced *F. japonica* are sister to each other (100% BS). Finally, *F. multinervis* is sister to the clade containing *F. engleriana* and *F. japonica* (moderate support, 73% BS), although its sister species remain undetermined.

## 4. Discussion

### 4.1. Intraspecific Variation and Plastome Evolution in Fagus

In the present study, we characterized two complete plastomes of the Japanese endemic *F. japonica*, which represents subgenus *Engleriana*, for the first time. These two plastomes showed very high similarity (i.e., 99.98% identity), with few indels and substitutions. Interestingly, sequencing of the second accession of *F. crenata* (subgenus *Fagus*), another species endemic to Japan, albeit with a much broader geographical distribution, revealed numerous indels and substitutions. These results indicate that *F. crenata*, which appears to have diverged much earlier than *F. japonica*, may show much greater intraspecific plastome variation. Since the two accessions of *F. japonica* were sampled from geographically close areas, this species may show much smaller variation than *F. crenata*. Therefore, this result warrants further confirmation through more extensive geographical sampling. Whole plastomes of three accessions of *F. multinervis*, which is endemic to Ulleung Island, from geographically different areas of the island showed very little variation. One frameshift insertion in *atpB*, resulting in early termination, one synonymous substitution in *psbM*, and one nonsynonymous substitution in *ccsA* were detected within *F. multinervis* plastomes. Similarly, very little variation in plastomes (i.e., just one haplotype) has been reported based on the *trnH*/*psb*A intergenic region, perhaps due to repeated colonization from a narrow source with a geographically structured chloroplast haplotype and a single long-distance seed dispersal event for the progenitor of *F. multinervis* [51]. Since very little variation was found in plastomes of *F. multinervis*, it is necessary to survey five highly variable regions within *Fagus* identified in current study (i.e., *ndhD/psaC*, *ccsA*/*ndhD*, *trnS*/*trnG*, *ycf1*, and *ndhF*/r*pl32*) to uncover the species’ phylogeographic structure on Ulleung Island.

Similar to the plastomes of several other congeners of Fagaceae [34,52], the East Asian species of *Fagus* tested in the present study had highly conserved plastomes, with no structural variations and content rearrangements. In addition, codon usage patterns of the five whole plastomes of East Asian *Fagus* species, including one plastome each of *F*. *crenata*, *F*. *engleriana*, and *F*. *multinervis* and two plastomes of *F*. *japonica*, were comparable. In these plastomes, codon usage was biased toward high RSCU values of U and A at the third codon position. Similar patterns have been observed in other angiosperm [53] and algal [54] lineages. Prediction of RNA editing sites in the five East Asian *Fagus* plastomes obtained consistent results with previous reports [55,56,57]. The *ndhB* (10 sites) had the highest number of potential editing sites, followed by *ndhD* (7 sites), in *F. japonica* and *F. multinervis*, whereas *rpoC2* had an exceptionally high number of editing sites (13 sites) in the single-stemmed *F. crenata*. The most frequent conversion was serine to leucine, and most RNA editing sites (82.3%) increased protein hydrophobicity.

Plastomes of all *Fagus* congeners in East Asia and Europe showed pseudogenization of three protein-coding genes, namely *infA*, *rpl22*, and *rps16*. While *infA* is intact in *Quercus*, the sister lineage of *Fagus*, it is lost in *Castanea*. Similarly, *infA* is lost in all studied *Fagus* species, indicating independent loss of this gene within Fagaceae [34]. Within Fagaceae, *rps16* became a pseudogene in *Fagus* but not in its sister lineages *Castanea* and *Quercus*. The *rpl22* became a pseudogene in all studied *Castanea*, *Quercus*, and *Fagus* species within Fagaceae, representing family synapomporphy. Based on comparative plastome analyses among members of the related order Malpighiales, Menezes et al. [58] reported the presence or absence of three protein-coding genes (*infA*, *rpl32*, and *rps16*) and two pseudogenes (*ycf1* and *rps19*). *infA* was present or absent in the plastome within the families of the order Malpighiales. The *rps16* and *rpl32* were independently lost in Violaceae and Salicaceae within this order. Given the evidence that some genes (*infA*, *rpl32*, and *rps16*) absent from the plastome in certain taxa are transferred to the nuclear genome in angiosperms [59,60,61], whether the three genes absent from the plastome of *Fagus* species were transferred to another genome or were completely lost should be investigated [62,63,64].

Most genes of the plastome evolve under purifying selection due to functional limitations during the course of evolution [65,66,67,68]. In the five *Fagus* plastomes studied, nearly 7.8% of protein-coding genes, including two photosynthesis-related genes encoding NADH-dehydrogenase subunits (*ndhD* and *ndhJ*), two genes encoding DNA-dependent RNA polymerase (*rpoB* and *rpoC2*), one self-replication-related gene encoding the ribosomal small subunit (*rps16*), and two unknown genes (*ycf1* and *ycf2*), were under positive selection pressure. Similar sets of genes (*ndhA*, *ndhK*, *petA*, and *ycf1*) were found to be under positive selection in the sister lineage *Quercus* [52]. Positive selection of genes encoding NADH-dehydrogenase and ribosomal complex has also been reported in other angiosperms, such as *Iodes* (Icacinaceae) and *Citrus* (Rutaceae) [69,70]. Moreover, positive selection of *ycf1* and *ycf2* has been suggested in *Iodes* (Icacinaceae [69]), *Panax* (Araliaceae [67]), and *Sileneae* (Caryophyllaceae [65]). Therefore, genes that protect plants from excess light and high temperature, such as NADH-dehydrogenase and ribosomal complex genes, may be positively selected in East Asian *Fagus* species [71,72]. Positive selection is considered to be indicative of adaptation to environmental changes, ecological niches, or coevolutionary processes [73,74]. Therefore, we speculate that the plastomes of *Fagus* species have contributed to their divergence and adaptation to temperate mixed deciduous forests in East Asia, although this topic warrants further research.

Identification and application of a highly variable or hotspot region in the plastome can achieve better resolution among closely related species or recently radiated groups. Recently, several hotspot regions, including genic and non-coding regions, across whole plastomes were reported based on the comparison between *F*. *crenata* (subgenus *Fagus*) and *F*. *engleriana* (subgenus *Engleriana*) [32]. The study found substantially lower pairwise nucleotide differences (p-distances) between the two species of *Fagus* than between those of its sister genus *Quercus* (0.0018 vs. 0.0042) [32]. In addition, Worth et al. [32] identified six highly variable regions (in decreasing order of variability, *ndhD*/*psaC*, *ndhI*/*ndhH*, *trnV*, *rpl32*, *trnG*/*psbfM*, and *psbK*/*psbI*) between two *Fagus* species. Sampling of additional East Asian *Fagus* species and their comparison with European species in the present study revealed five highly variable regions (in decreasing order of variability, *ndhD*/*psaC*, *ccsA*/*ndhD*, *trnS*/*trnG*, *ycf1*, and *ndhF*/*rpl32*). Thus, the *ndhD*/*psaC* intergenic region is the only and the most variable region in genus *Fagus* based on both the present and previous studies. As closely related species and conspecific accessions were included to identify hotspots, previously identified regions (*ndhI*/*ndhH*, *trnV*, *rpl32*, *trnG*/*psbfM*, and *psbK*/*psbI*) [32] showed significantly lower *Pi* values (<0.00492 for *ndhI*/*ndhH*) than the four regions (*ccsA*/*ndhD*, *trnS*/*trnG*, *ycf1*, and *ndhF*/*rpl32*; *Pi* > 0.008) identified in the present study. Therefore, the five highly variable regions identified in this study can be effective chloroplast DNA markers for population genetic and phylogeographic studies of *Fagus* species. When these hotspots were compared to those in the closest sister lineage *Quercus*, some regions (e.g., *ycf1* and *ccsA*/*ndhD*) were consistently recognized as hotspots between the two genera, while others (e.g., *rpl22*, *rps16*, *trnR*/*atpA*, and *trnM*/*atpE*, among others) were identified in *Quercus* alone, indicating that these regions may serve as effective taxon-specific phylogenetic and DNA barcoding markers.

### 4.2. Phylogenetic Position and Relationship of Fagus multinervis on Ulleung Island

Since there are no *Fagus* populations in the Korean Peninsula, the origin and evolution of *F. multinervis* endemic to Ulleung Island with respect to its close relatives *F. engleriana* and *F. japonica* have long been controversial. *F. engleriana* is distributed in western China (Sichuan, Guizhou, and western Hubei; above 1200 m elevation) and eastern China (southern Anhui and northwestern Zhejiang; between 900 and 1700 m elevations) [5,9]. Based on similar cupule characteristics between *F. engleriana* and *F. multinervis* (i.e., the base of cupules is covered with leaf-like bracts), Shen [5] treated *F. multinervis* as a conspecific of *F. engleriana* in China, indicating disjunct distribution in western China, Central China, and Ulleung Island, Korea. Meanwhile, based on the similarity of leaves, anthers, and pollen, Lee [10] considered *F. multinervis* an ecological variant of *F. japonica*, which is distributed in central and southern Japan. Some authors have also treated *F. multinervis* as a synonym of *F. engleriana* [5,11], while others have treated it as a distinct species based on floristics, allozymes, and comparative descriptions [12,13,15]. Based on whole plastomes of *Fagus* species, we hoped to determine the closest relative of *F. multinervis* on Ulleung Island. Unfortunately, however, even with whole plastome data, we could not determine the closest species of *F. multinervis*. The plastome phylogenomic tree suggested that genus *Fagus* is monophyletic (100% BS support) and that monophyletic subgenus *Engleriana* is deeply nested within subgenus *Fagus* (Figure 6). Moreover, *F. crenata*, which is endemic to Japan, diverged first, followed by the European species *F. sylvatica*. Within subgenus *Engleriana*, *F. multinervis* is a distinct and monophyletic clade and is sister to the clade containing two closely related species, *F. japonica* and *F. engleriana*. Based on combined plastid sequence data, Oh et al. [9] demonstrated that *F. japonica* accessions cultivated in the Arnold Arboretum (USA) formed a clade with *F. crenata* cultivated in the Tsukuba Botanical Garden (Japan) and Arnold Arboretum. Conversely, *F. japonica* cultivated in the Arnold Arboretum was sister to the clade containing *F. engleriana* and *F. multinervis* on Ulleung Island and was, in turn, sister to the clade containing *F. engleriana*, *F. lucida*, *F. longipetiolata*, and *F. japonica*. These relationships imply plastid capture via hybridization or introgression, both of which are known frequent processes in Fagaceae [75,76]. The nuclear *LEAFY* phylogeny, however, is highly unresolved in terms of the phylogenetic position of *F. multinervis*; the strongly supported clade (100% BS and posterior probability (PP) 1.0) includes monophyletic *F. multinervis* (72% BS and PP 1.0), monophyletic *F. engleriana* (65% BS and PP 1.0), and non-monophyletic *F. japonica*. Oh et al. [9] further argued that given the patterns of phylogenetic incongruence between plastid and nuclear phylogenies and certain shared morphological traits, *F. multinervis* could be a hybrid between *F. engleriana* in China and *F. japonica* in Japan. The divergence of two close relatives *F. engleriana* and *F. japonica* was estimated to be 9.3 to 10.1 Ma, but the fossils of *Fagus* in the Korean Peninsula date back to the early Miocene (16.8 Ma) [6,77]. Given the very young age of Ulleung Island (ca. 1.8 Ma), it is difficult to determine the precise timing of the origin of *F. multinervis* on this island. If *F. multinervis* is indeed a hybrid of *F. engleriana* and *F. japonica*, this hybridization event might have occurred between 10 and 2 Ma on the Korean mainland or the Japanese archipelago, and the ancestor of *F. multinervis* became extinct from these regions later or simultaneously dispersed to Ulleung Island as early as 1.8 Ma. Alternatively, a different position of *F. multinervis* on plastid and nuclear trees [9] may be obtained due to lack of sufficient resolution and/or incomplete lineage sorting of nuclear genes, given their greater coalescence time than that of plastid genes. Although previous studies do not support the direct divergence of *F. multinervis* from *F. engleriana* in China or *F. japonica* in Japan, this possibility cannot be completely ruled out. If these hypotheses are supported, the shared morphological traits between *F. multinervis* and *F. engleriana* or *F. japonica* could be a result of shared ancestral traits (symplesiomorphy) or convergent evolution. Lack of determination of sister species and the precise geographical origin of *F. multinervis* was further confirmed by the present study. Nonetheless, the present results, consistent with previous reports [78], strongly support the recognition of *F. multinervis* as a distinct species endemic to Ulleung Island. As previously suggested [9], extensive sampling of *F. engleriana* populations from China and *F. japonica* populations from Japan as well as phylogenomic and molecular dating analyses are warranted to gain insight into the origin and evolution of *F. multinervis* on Ulleung Island.

## Figures and Tables

**Table 1:** Summary of characteristics of four *Fagus* plastomes in eastern Asia.

Taxa	*F. crenata*	*F. japonica7-1*	*F. japonica10-1*	*F. multinervis*
Total cpDNA size (bp)	158,227	158,163	158,193	158,348
GC content (%)	37.1%	37.1%	37.1%	37.1%
LSC size (bp)/GC content (%)	87,552/35.1%	87,590/35.1%	87,620/35.1%	87,659/35.0%
IR size (bp)/GC content (%)	25,873/42.7%	25,894/42.7%	25,893/42.7%	25,893/42.7%
SSC size (bp)/GC content (%)	18,929/31.1%	18,785/31.2%	18,787/31.2%	18,903/31.1%
Number of genes	131	131	131	131
Number of protein-coding genes	82	82	82	82
Number of tRNA genes	37	37	37	37
Number of rRNA genes	8	8	8	8
Number of duplicated genes	17	17	17	17
Accession Number	MT762292	MT762294	MT762295	MT762296

LSC: Large single-copy region, IR: Inverted repeat, SSC: Small single-copy region.

**Table 2:** Log-Likelihood values of the site-specific models, with detected sites having dN/dS values > 1.

Gene Name	Models	np	ln L	Likelihood Ratio Test *p*-Value	Positively Selected Sites
*ndhD*	M8	15	−2087.368596	0.000000130	385 T 0.951 *
	M7	13	−2103.224804		
*ndhJ*	M8	15	−670.195417	0.000000009	151 F 0.995 **
	M7	13	−688.698395		
*rpoB*	M8	15	−4347.768637	0.000000009	248 Q 0.959 *
	M7	13	−4372.590740		
*rpoC2*	M8	15	−5635.415430	0.000000000	748 Y 0.984 *,1338 I 0.983 *
	M7	13	−5656.937844		
*rps16*	M8	15	−242.458940	0.000000720	1 M 0.995 **
	M7	13	−256.602491		
*ycf1*	M8	15	−7160.045295	0.000000000	372 K 1.000 **, 373 T 1.000 **, 842 F 0.991 **, 905 P 0.991 **, 1066 R 0.992 **, 1142 F 0.991 **, 1284 N 1.000 **, 1286 D 0.991 **, 1359 F 0.999 **
	M7	13	−7218.177588		
*ycf2*	M8	15	−9220.322295	0.000000000	388 T 0.982 *
	M7	13	−9248.972947		

* *p* < 0.05; ** *p* < 0.01. np represents the degree of freedom.

## Supplementary Materials

The following are available online at https://www.mdpi.com/2073-4425/11/11/1338/s1, Table S1. The predicted RNA editing sites in the complete plastome of five Fagus species in eastern Asia.
